# The Neighborhoods Study: Examining the social exposome in Alzheimer's disease and related dementias

**DOI:** 10.1002/alz.70810

**Published:** 2025-11-13

**Authors:** Grace C. George, Sarah A. Keller, Erin Abner, Sara Adar, Michael L. Alosco, Liana G. Apostolova, Kelly Bakulski, Lisa L. Barnes, James R. Bateman, Stuart Batterman, Thomas G. Beach, Barbara B. Bendlin, David A. Bennett, Tobey J. Betthauser, James Brewer, William Buckingham, Carmen I. Carrión, Joshua Chodosh, Suzanne Craft, Raina Croff, Anthony Fabio, Sarah Tomaszewski Farias, Eva L. Feldman, Felicia Goldstein, Stephen A. Goutman, Gina Green‐Harris, Victor Henderson, Thomas K. Karikari, Julia Kofler, Anna Kucharska‐Newton, Melissa Lamar, Serggio Lanata, Rebecca J. Lepping, Jennifer Lingler, Samuel Lockhart, Jonathan Mahnken, Karyn Marsh, Jomol Mathew, Adam P. Mecca, Oanh Meyer, Bruce Miller, Jill Morris, Judith Neugroschl, Maureen K. O'Connor, Henry Paulson, Richard J. Perrin, Corinne Pettigrew, Aimee Pierce, W. Ryan Powell, Saiju Pyarajan, Cyrus A. Raji, Eric Reiman, Shannon Risacher, Robert Rissman, Patricia Rodriguez Espivnosa, Mary Sano, Andrew J. Saykin, Geidy E. Serrano, Vikas Singh, Anja Soldan, David Sultzer, Christopher H. van Dyck, Rachel Whitmer, Thomas Wisniewski, Randall Woltjer, Menggang Yu, Carolyn W. Zhu, Amy J. H. Kind

**Affiliations:** ^1^ Center for Health Disparities Research University of Wisconsin School of Medicine and Public Health UW Hospital and Clinics Madison Wisconsin USA; ^2^ Department of Epidemiology University of Kentucky College of Public Health Lexington Kentucky USA; ^3^ Departments of Epidemiology and Global Public Health University of Michigan School of Public Health Ann Arbor Michigan USA; ^4^ Department of Neurology Boston Medical Center Boston Massachusetts USA; ^5^ Boston University Alzheimer's Disease Research Center BU CTE Center Department of Neurology Boston University Chobanian & Avedisian School of Medicine Boston Massachusetts USA; ^6^ Department of Anatomy & Neurobiology Boston University Chobanian & Avedisian School of Medicine Boston Massachusetts USA; ^7^ Indiana Alzheimer's Disease Research Center Indiana University School of Medicine Indianapolis Indiana USA; ^8^ Rush Alzheimer's Disease Center Rush University Medical Center Chicago Illinois USA; ^9^ Departments of Neurology and Psychiatry Wake Forest University School of Medicine Winston‐Salem North Carolina USA; ^10^ Department of Global Public Health University of Michigan School of Public Health Ann Arbor Michigan USA; ^11^ Department of Pathology Banner Sun Health Research Institute Sun City Arizona USA; ^12^ Department of Medicine University of Wisconsin–Madison School of Medicine and Public Health Madison Wisconsin USA; ^13^ Wisconsin Alzheimer's Disease Research Center University of Wisconsin–Madison School of Medicine and Public Health Madison Wisconsin USA; ^14^ Department of Neurosciences University of California San Diego La Jolla California USA; ^15^ Department of Neurology Yale School of Medicine New Haven Connecticut USA; ^16^ Department of Geriatrics New York University Grossman School of Medicine New York New York USA; ^17^ Department of Neurology Oregon Health and Science University School of Medicine Portland Oregon USA; ^18^ Department of Epidemiology and Clinical and Translational Sciences University of Pittsburgh School of Public Health Pittsburgh Pennsylvania USA; ^19^ Department of Neurology University of California Davis, School of Medicine Davis California USA; ^20^ Department of Neurology University of Michigan Ann Arbor Michigan USA; ^21^ Department of Neurology Emory University School of Medicine Atlanta Georgia USA; ^22^ Department of Neurology University of Michigan School of Medicine Ann Arbor Michigan USA; ^23^ Department of Epidemiology and Population Health Stanford University School of Medicine Stanford California USA; ^24^ Department of Psychiatry University of Pittsburgh School of Medicine Pittsburgh Pennsylvania USA; ^25^ Department of Epidemiology University of North Carolina at Chapel Hill Chapel Hill North Carolina USA; ^26^ Department of Neurology University of California San Francisco San Francisco California USA; ^27^ Department of Neurology University of Kansas Medical Center Kansas City Kansas USA; ^28^ Univeristy of California Davis School of Nursing Sacramento California USA; ^29^ Department of Biostatistics & Data Science University of Kansas Medical Center Kansas City Kansas USA; ^30^ Department of Neurology New York University Grossman School of Medicine New York New York USA; ^31^ Department of Psychiatry Yale School of Medicine New Haven Connecticut USA; ^32^ Department of Internal Medicine Icahn School of Medicine at Mt. Sinai New York New York USA; ^33^ Department of Pathology and Immunology Washington University School of Medicine St. Louis Missouri USA; ^34^ Department of Neurology Johns Hopkins University School of Medicine Baltimore Maryland USA; ^35^ Department of Radiology Washington University School of Medicine St. Louis Missouri USA; ^36^ Department of Psychiatry Banner Sun Health Research Institute Sun City Arizona USA; ^37^ Department of Psychiatry Icahn School of Medicine at Mt. Sinai New York New York USA; ^38^ School of Medicine University of California, Irvine Irvine California USA; ^39^ Department of Neurology Pathology, and Psychiatry New York University Grossman School of Medicine New York New York USA; ^40^ Pathology & Laboratory Medicine Oregon Health and Science University School of Medicine Portland Oregon USA; ^41^ Biostastics University of Michigan School of Public Health Ann Arbor Michigan USA; ^42^ Department of Geriatrics and Palliative Medicine Icahn School of Medicine at Mt. Sinai New York New York USA

**Keywords:** adverse social exposome, Alzheimer's disease and related dementias, Area Deprivation Index, brain health, exposome, health disparities, life‐course exposures, neighborhood disadvantage, neuropathology, social determinants of health, The Neighborhoods Study

## Abstract

**INTRODUCTION:**

The Neighborhoods Study (TNS) is a novel investigation of adverse social exposome and brain health leveraging 22 Alzheimer's Disease Research Centers (ADRCs). TNS aims to understand if the adverse social exposures increase Alzheimer's disease and related dementias (ADRD) risk.

**METHODS:**

TNS uses innovative methods to determine lifetime addresses of living (*n* = ≈ 3116) and brain bank cohorts (*n* = ≈ 8637). Addresses are linked to time‐concordant adverse social exposome using the Area Deprivation Index (ADI) and summarized over time. Brain health measures are provided by the National Alzheimer's Coordinating Center.

**RESULTS:**

We highlight a general overview and methodology of TNS. Data collection is ongoing; however, preliminary findings indicate that the adverse social exposome is related to ADRD biomarkers, neuropathology, and cognitive function.

**DISCUSSION:**

TNS is the largest study of adverse social exposome and ADRD, using the ADRC network to build robust scientific consortia. Its findings will inform ADRD interventions, precision medicine, and policy.

**Highlights:**

- The Neighborhoods Study (TNS) investigates adverse social exposome and brain health.
- TNS is a collaboration among 22 Alzheimer's Disease Research Centers.
- TNS will give insight on environmental and exposomal factors which may be modifiable.
- Participant lifetime addresses are linked to temporal adverse social exposome metrics.
- This study's findings will inform precision approaches to mitigate dementia risk.

## 1 BACKGROUND

Alzheimer's disease and related dementias (ADRD) affect 55 million people worldwide,^1^ and certain high‐risk populations are disproportionately impacted by this disease. To most effectively prevent and treat ADRD, a much deeper understanding of the factors that drive these higher risks is needed, with increasing attention paid to the many environmental disease‐driving forces which may be modifiable.^2^ The exposome is a conceptual framework to study the holistic experience of the individual, and how cumulative and individual experiences contribute to health and disease.^3^ The life‐course social exposome is a particularly important factor within exposome research, encompassing external social factors to an individual including, but not limited to, neighborhood disadvantage, occupational environment, and social determinants of health. These perspectives will allow researchers to ask mechanistic questions on health disparities outcomes and can reveal life‐course patterns and sensitive periods that may be instrumental in the development of (or resilience to) ADRD. Recent ADRD work suggests that a strong association exists between the adverse social exposome and poorer brain health.^4^, ^5^, ^6^, ^7^ Yet only a few deeply biologically phenotyped brain health studies have linked social exposures to their existing clinical and biological data, and most of these offer only limited life‐course perspectives.^8^ Understanding early‐, mid‐, and late‐life exposures will help unlock understanding of key ADRD risks, focus interventions, and inform preventive interventions for the future.

Some multi‐site studies have begun to address disparities^9^ and brain imaging in ADRD,^10^ but have not considered the life‐course exposome and lacked comprehensive brain tissue analysis and outcomes of neurodegenerative disease. While these studies have shaped the current understanding of ADRD, they raised additional questions on the nature of associations present, such as whether precise measures of adverse exposures over the life course, like neighborhood disadvantage, improve predictive power for ADRD outcomes and severity.

**FIGURE 1 alz70810-fig-0001:**
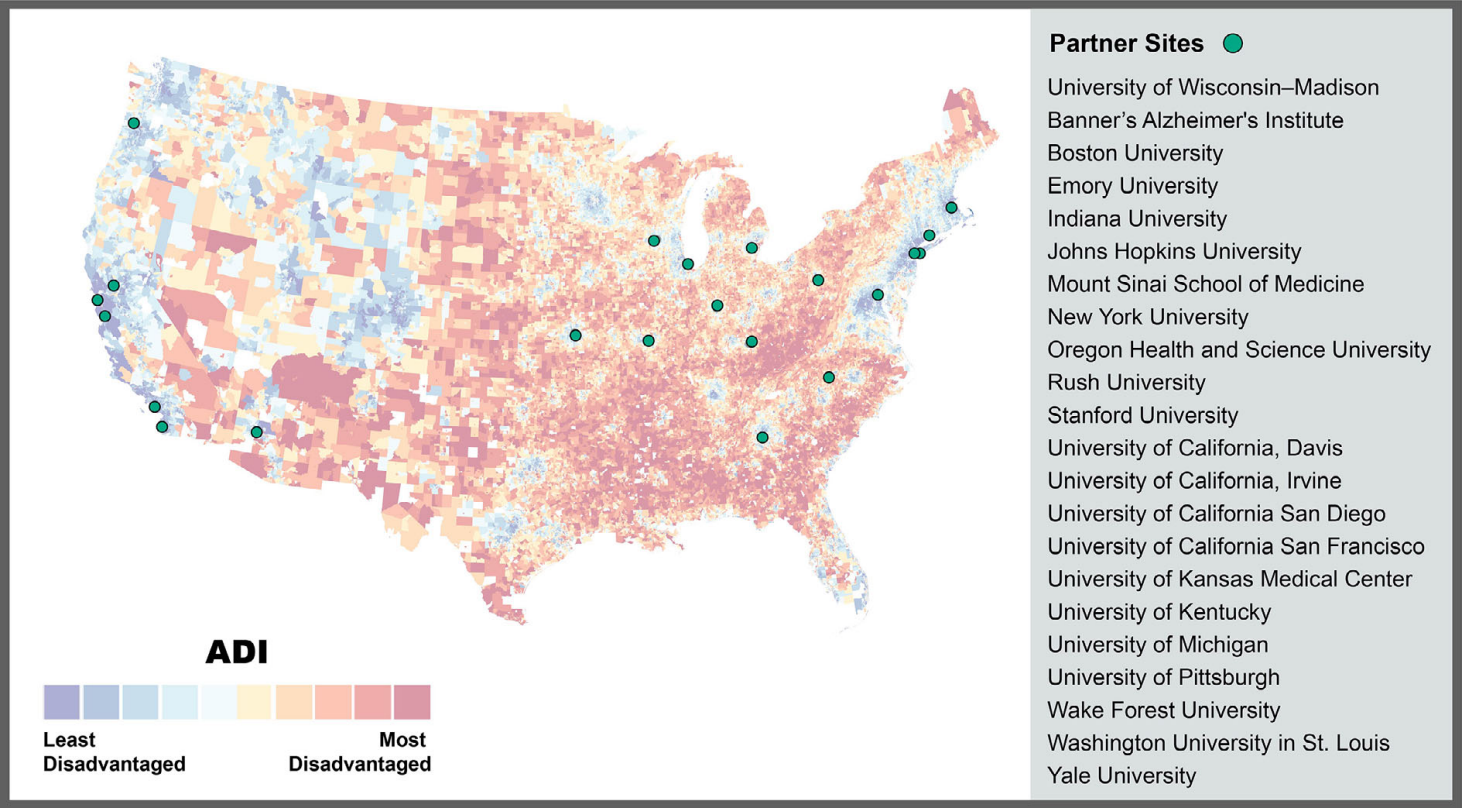
National ADI Deciles and The Neighborhoods Study Locations. National ADI for the United States with the 22 Alzheimer's Disease Research Centers overlayed (represented as green circles). National decile colors from low ADI (blue) to high ADI (red) indicating least to most disadvantaged and are measured to the census block group. *Source*: free, publicly available at https://www.neighborhoodatlas.medicine.wisc.edu/. ADI, Area Deprivation Index.

With this concept in mind, we launched The Neighborhoods Study (TNS; R01AG070883) to establish feasible new ways to connect the social exposome to biological markers of the ADRD brain to measure the adverse social exposome, indexed by neighborhood disadvantage, over the life course, and to identify the key effect mediators and moderators involved. TNS leverages 22 sites across the National Institutes of Health (NIH)‐funded Alzheimer's Disease Research Center (ADRC) network and their brain banks (Figure 1).

**RESEARCH IN CONTEXT**

1. **Systematic review**: Literature was reviewed using standard sources, such as PubMed. Previous studies of life‐course adverse social exposome have found associations between these factors and Alzheimer's disease and related dementias (ADRD) and are referenced in the article.
2. **Interpretation**: This article describes the rationale, design, and methodology of The Neighborhoods Study (TNS), a multisite study including 22 National Institutes of Health–funded Alzheimer's Disease Research Centers. This is the largest study of its kind connecting the social exposome to biological markers of ADRD.
3. **Future directions**: This study will provide novel infrastructure key to forwarding ADRD exposome science, while building a scientific consortium toward new knowledge. Its findings may inform new approaches that can be used by clinicians, researchers, and policy makers to help mitigate ADRD.

The TNS and ADRC cooperation created the largest study of its kind on the social exposome and its relation to ADRD pathogenesis. This work would not have been possible without the pre‐existing infrastructure and resources of the national ADRC network and the National Alzheimer's Coordinating Center (NACC), which includes up to 25 years’ worth of data collection on brain and cognitive health.^11^ However, the relationship between adverse social exposome factors and ADRD neuropathology requires a novel infrastructure to create a well‐phenotyped history of ADRC participants and donors. To accomplish this, we combined survey (for living participants) and archival data (i.e., census records, marriage certificates, etc., for brain donors) to establish a well‐documented participant specific residential history over[Fig alz70810-fig-0001] the life course.^12^ This allows our team to measure aspects of a person's current and past situational and environmental context and connect it with their brain health outcomes, as assessed by biomarkers or at autopsy. We can do this due to our highly knowledgeable team of scientists, historians, and geography specialists who are unlike any other group in the country.

**FIGURE 2:**
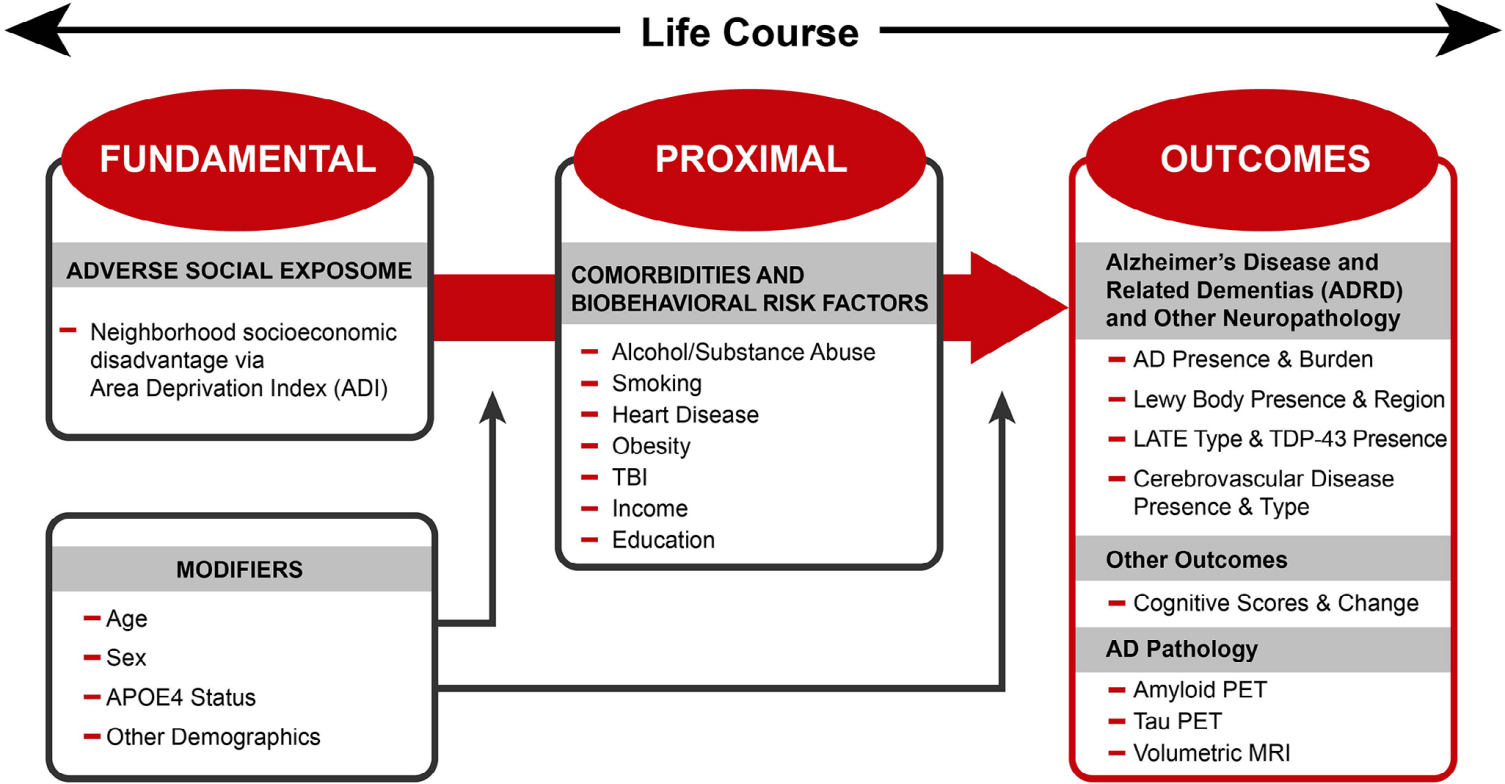
TNS conceptual model. Fundamental Causes Framework^13^ of socio‐biological pathways impacting brain outcomes. The life course, including lifetime dosage, timing accumulation, and an assessment of sensitive developmental stages including childhood, young adult, midlife, and older adult will be used to assess outcomes via ADI. AD, Alzheimer's disease; ADI, Area Deprivation Index; *APOE*, apolipoprotein E; LATE, limbic‐predominant aging‐related TDP‐43 encephalopathy; MRI, magnetic resonance imaging; PET, positron emission tomography; TBI, traumatic brain injury; TDP‐43, tau DNA‐binding protein 43; TNS, The Neighborhoods Study.

Herein, we offer a broad overview of TNS design and methodology, with a focus on the developed novel infrastructure and scientific capacity growth targeted toward catalyzing ADRD social exposome studies. We provide key insights on how the ADRC national network made this study possible. The TNS grounding hypothesis posits that earlier and more frequent life‐course exposures to adverse social exposome factors increases ADRD risk (Figure 2). The first funding period of the study focuses on neighborhood disadvantage, recognizing that many other social exposome factors will need to be assessed in the future.

## 2 METHODS

### 2.1 Project oversight and management

Led by the University of Wisconsin (UW) School of Medicine and Public Health in Madison, Wisconsin, USA, TNS is a multidisciplinary collaboration among 22 ADRCs, with an established group of site primary investigators (PIs) and co‐investigators (Co‐Is) offering unique perspectives and research specialties. This study also is directed by TNS Steering Committee to help with oversite and decision making.

**FIGURE 3:**
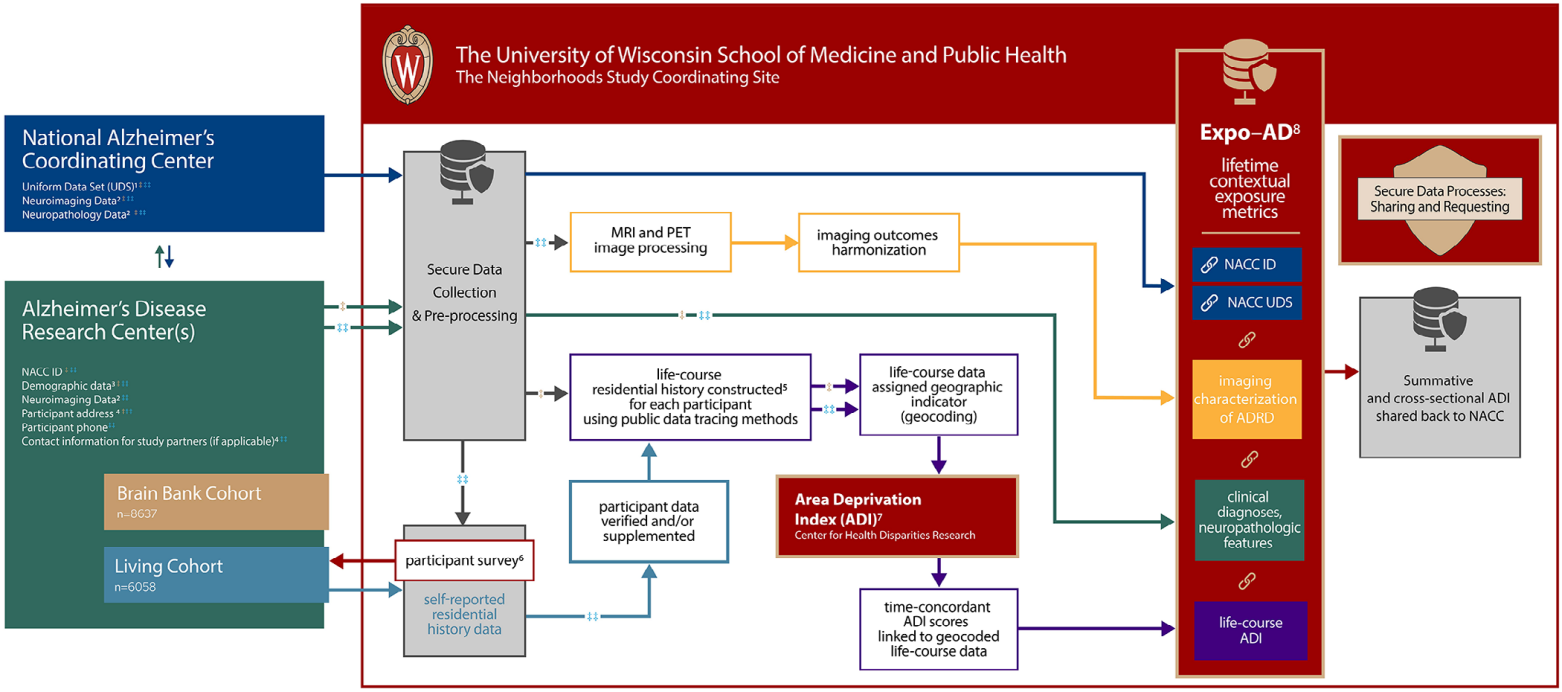
The Neighborhoods Study methods diagram. Description of TNS workflow from left to right, describing how data from NACC and ADRCs flow through UW School of Medicine and Public Health processing through secure data sources. Life‐course residential histories are geocoded to generate life‐course ADI measures for each participant, which are de‐identified and disseminated through NACC. ‡ Brain Bank Donor Cohort. ‡‡ Living Cohort. The Neighborhoods Study Methods Diagram. ^1^UDS data elements to be used in this study include participants NACC IDs, demographic characteristics (name, age, sex and race/ethnicity, education, income, occupation, decedent next of kin name and address, behavioral risk factors, physiological indicators, social risk factors, objective measures of cognitive function and clinical diagnosis). ^2^Neuroimaging data include MRI, amyloid PET411, and tau PET scans. Donor *ante mortem* raw neuroimaging data (where available). ^3^Demographic data collected from sites include name, age, sex and race/ethnicity, education, income, occupation, decedent next of kin name and address if available, behavioral risk factors, physiological indicators, social risk factors, objective measures of cognitive function and clinical diagnosis. ^4^After IRB approval and the completion and execution of a DUA, eligible participants are identified and consent to be contacted in one of two ways, according to local ADRC policies and practices: direct contact or opt in. ^5^The Historical Archive Research Team uses public data tracing methodologies based on standard genealogical research and unstructured records abstraction methods to create detailed life‐course residential histories for each subject. See Melcher et al.^12^ ^6^The study uses mail survey methodology as its means of data collection for living cohort participants. The surveys are administered securely by the UW–Madison Survey Center. ^7^ To calculate historical ADI scores, we use publicly available data, including US Census and American Community Survey data. ADIs have been constructed to cover the years 1910 to the present, weighting the poverty, education, housing, and employment indicators for a given time period of interest using factor score coefficients derived from factor analysis. ^8^Expo‐AD: developed under The Neighborhoods Study (NIH/NIA R01AG070883) administrative supplement, Expo‐AD offers a novel collaborative infrastructure of contextual exposure for future social‐biological phenotypic evaluation, providing a potential pathway to new therapeutics. Directly responsive to the NIA mission, Expo‐AD provides a shared framework of contextual exposure for social‐biological phenotypic assessment, linking metrics of lifetime contextual exposure to well‐characterized, harmonized ADRD‐focused bioresources, including neuroimaging and brain bank data. Expo‐AD is unparalleled in its harmonization of clinical, neuropathologic, and imaging characterization of ADRD and ability to provide a foundation for robust multi‐factor phenotypic risk assessment of social‐biological interactions and their mechanisms, including future "omics" work. AD, Alzheimer's disease; ADI, Area Deprivation Index; ADRC, Alzheimer's Disease Research Center; ADRD, Alzheimer's disease and related dementia; DUA, data use agreement; IRB, institutional review board; MRI, magnetic resonance imaging; NACC, National Alzheimer's Coordinating Center; NIA, National Institute on Aging; PET, positron emission tomography; TNS, The Neighborhoods Study; UDS, Uniform Data Set; UW, University of Wisconsin.

All PIs and Co‐Is convene regularly to discuss operations, analyses, data, strategy, and reporting of the study. Each site PI is responsible for facilitating and processing data use agreements, institutional review boards (IRBs), and fulfilling other administrative requirements. NACC, a multi‐site national consortium, allows for the harmonization of clinical, neuropathologic, and imaging characterization of ADRD. Workflow and a methodology diagram can be found in Figure 3.

### 2.2 Study participants and donor samples

TNS consists of a living cohort (*n* = 3116 expected from 18 of the 22 sites) and the brain bank cohort (*n* = 8637 expected from 21 of 22 sites) as of May 2025. All participants were required to have a NACC participant ID, full name, and at least one address to be eligible. Data use agreements between UW and each participating institution, customized for each US state's privacy laws, were created to share identifiable participant information required for residential history construction. All IRB activities were conducted through Western Institutional Review Board (W)IRB‐Copernicus Group (WCG) servers (https://www.wcgclinical.com/), which assists researchers and studies with administration and maintenance of study protocols and documents. They help provide review and approval of the protocol and study documents among the 22 sites (WCG IRB tracking ID 20212477).

For the living cohort, each eligible participant was identified by their ADRC and contact information was shared with UW to allow for paper surveys to be mailed by the UW Survey Center to each identified participant. Information sharing followed standard ADRC‐specific processes for each site. Each site encouraged their participants to fill out the mailed paper residential history survey using a standard protocol of activities (for more information, see below). Survey materials were available in English and Spanish.

For the brain bank cohort, all participants included were brain donors with neuropathology assessments reported in the NACC Neuropathology Data Set.^14^ Consent followed each site's protocols for brain donation.

### 2.3 Exposomal data collection: residential history

Living cohort participants were mailed surveys that asked about their residential histories and their sociodemographic characteristics including race, ethnicity, years of education, and sex using a standard protocol of contacts and reminders. Completed surveys were returned to the UW coordination center. All addresses were then cleaned and verified for geocoding.

For the brain donor cohort, each participating ADRC sent the NACC ID, birth date, death date, name, and last known address to UW for eligible decedent brain donors via secure data transfer protocols. The UW team used this information as a starting point to query publicly available records to construct each participant's residential history. Publicly available document sources include obituaries, birth certificates, and newspaper articles. All addresses were then checked and cleaned for optimal geocoding. Information on deriving life‐course residential histories from public data for brain donors has been reported previously, see Melcher et al.^12^

### 2.4 Neighborhood disadvantage life‐course dosage and timing

Each address within a residential history is geocoded, a way to provide geographical coordinates to a location, that is, residential address, and linked to a time‐concordant measure of adverse social exposome, the Area Deprivation Index (ADI) from 1930 until today.^15^ The ADI is a validated measure of neighborhood disadvantage in the United States, and the modern ADI consists of 17 different indicators, derived from the US Census and American Community Survey (ACS), that are related to poverty, education, housing, and employment indicators to characterize these social determinants within census block‐groups (i.e., “neighborhoods”).^15^ This enables the study of how and to what extent the life‐course exposome, based on residential histories and indexed by ADI, is related to ADRD. The ADI has been developed for both the modern ACS period (2009–present) as well as for the decennial periods from 1930 through 2000. While not fully comprehensive of the entire social exposome, having the ADI be a geolinked measure also allows other exposomal metrics tied to a geographic location, like proximity to clinical care, to be easily used in tandem. The ADI is free for the public to use and is being used by researchers, clinicians, corporations, and governments around the country https://www.neighborhoodatlas.medicine.wisc.edu/. Furthermore, the in‐depth archival histories created for each donor as part of this study will offer rich data resources for additional future social exposome measure assessment, including occupation, family structure, and other key factors.

### 2.5 Brain health outcomes and risk factors

Both the living and brain bank cohorts had markers of brain health and brain health risk factors drawn from the Uniform Data Set (UDS) and the Minimum Data Set (MDS) data centralized in the NACC database and from data provided by each site, including neuroimaging.

The brain bank cohort outcomes include measurements of ADRD pathology such as amyloid plaque and neurofibrillary tangles, cerebrovascular disease, Lewy body pathology, and frontotemporal lobar degeneration (FTLD), conditional on each ADRC's data collection and availability via NACC neuropathology dataset (Figure 2).^16^

### 2.6 Scientific consortium building for ADRD social exposome study

TNS, through its multi‐site research infrastructure activities, has yielded promising results for the future of this consortium. We continue to leverage the opportunities presented by this award to build and foster scientific community interested in the intersection of exposomal experiences and biological aging, with particular emphasis on collaboration, communication, and coordination. In addition to regular study team meetings, a convening event held at the 2024 Alzheimer's Association International Conference (AAIC) provided an opportunity to share findings, collaborate on data sharing processes, and share opportunities for engagement in committees and subgroups toward future production and collaboration. We plan on continuing this tradition biannually to accommodate new and young investigators.

We also produce The Neighborhoods Study Quarterly (TNSQ), a multi‐site newsletter delivered to PIs, site PIs, Co‐Is, key personnel, and members of the research and administrative teams involved in the study. The newsletter has continued to grow throughout the reporting period, with a circulation of 184 subscribers and a 69% open rate. The purpose of the newsletter is to facilitate connectivity, communication, and community across all 22 sites; disseminate study‐related updates, progress, successes, and opportunities; and showcase the efforts of those contributing to the project, including trainees and junior investigators.

Building on the success of the TNSQ, our team launched a secondary biweekly procedural‐focused newsletter, Neurons to Neighborhoods, committed to the specific needs of our TNS colleagues participating in the living cohort component of the study. With a smaller, targeted audience of 92 subscribers, the bespoke Living Cohort newsletter likewise is sent to a highly engaged research community. TNS continues to attract interest and attention widely, creating opportunities for future collaborations, proposals, and scientific products resulting from this work (see the next section for metrics of success).

## 3 RESULTS

As of May 2025, TNS exposomal data collection is ongoing. Current demographics for the brain bank study can be found in Table 1. For the brain bank study, all 8637 donors have geocoded ADI at time of death; currently, life‐course residential histories have been completed for approximately one third, with full completion anticipated in 2026. Brain bank life‐course ADIs have been appended to 54,711 addresses to create the sample's life‐course residential histories (95% of locations geocodable). The brain bank study cohort had an average (standard deviation, range) of 27.78 (9.81, 1–72) addresses found and which post‐processing covered ≈ 83% of their life course. Further, 25.78 (9.28, 1–72) residences were successfully geocoded per person and post‐processing covered ≈ 82% of their life course.

**TABLE 1:** Living and brain donor cohort demographics for currently enrolled and characterized participants.

Characteristic	Living cohort samples *N* = 3116	Brain bank sample *N* = 8637
Sex, no. (%)		
Female	1969 (63%)	4415 (51%)
Male	1147 (37%)	4222 (49%)
Year of birth, no. (%)		
Before 1900	0 (0%)	52 (0.6%)
1900–1909	0 (0%)	588 (6.8%)
1910–1919	1 (0.03%)	2157 (25%)
1920–1929	47 (1.5%)	2833 (32.8%)
1930–1939	486 (15.6%)	1695 (19.6%)
1940–1949	1292 (41.5%)	899 (10.4%)
1950–1959	961 (30.8%)	318 (3.7%)
1960 and later	329 (10.6%)	95 (1.1%)
Age at survey or death, no. (%)		
<65	495 (15.9%)	673 (7.8%)
65–69	377 (12.1%)	565 (6.5%)
70–74	501 (16.1%)	811(9.4%)
75–79	680 (21.8%)	1213 (14%)
80–84	565 (18.1%)	1649 (19.1%)
85–89	329 (10.6%)	1630 (18.9%)
≥ 90	169 (5.4%)	2082 (24.1%)
Missing	0 (0%)	14 (0.2%)
Race/ethnicity, no. (%)		
Black or African American	349 (11.2%)	328 (3.8%)
White	2489 (79.9%)	8104 (93.8%)
Hispanic or Latino	202 (6.5%)	235 (2.7%)
Asian or Pacific Islander	90 (2.9%)	60 (0.9%)
American Indian or Alaska native	29 (0.9%)	11 (0.1%)
Multiracial	124 (4%)	81 (1%)
Unknown or ambiguous	35 (1.1%)	53 (0.6%)
Education, no. (%)		
Less than high school	50 (1.6%)	839 (7.7%)
High school or GED	269 (8.6%)	1967 (22.8%)
Some college	553 (17.7%)	1529 (17.7%)
Bachelor's degree	813 (26.1%)	1844 (21.4%)
Post‐graduate degree	1426 (45.8%)	2347 (27.2%)
Unknown	5 (0.2%)	111 (1.3%)
*APOE* ε4 presence % (assessed *N*)	33% (2276)	40% (6757)

*Note*: For *APOE* ε4 numbers indicate presence (%) of total assessed (N). Percentages may not be 100% due to rounding. Participant and donor demographics of the living and brain bank cohorts.

Abbreviations: *APOE*, apolipoprotein E; GED, General Educational Development.

Our team is currently collecting life‐course residencies for the living cohort and will soon start geocoding and creating life‐course ADIs. We expect to receive all addresses for the living cohort in late 2025.

Preliminary findings and early studies have found that exposure to more adverse social exposome indexed by higher ADI has been related to greater neurodegeneration, lower cognitive function, less favorable ADRD biomarkers measurements, and more advanced *post mortem* ADRD neuropathologies.^4^, ^5^, ^6^, ^7^ TNS has supported > 50 publications, 18 NIH studies, and provided essential data and unique infrastructure to 10 early career researchers who have based their own grants directly on the study's resources. We owe this through the tremendous assistance of ADRCs, NACC, and our willing PIs and Co‐Is that prioritize collaboration and support the larger mission of tackling health disparities in ADRD. We are encouraged by these important and novel findings, and the promise of this unique collaborative scientific and exposome infrastructure to enable key progress across the ADRD exposome field. Key TNS data will be shared widely through NACC to catalyze future study.

## 4 DISCUSSION

TNS provides a rich and unique collection of social exposome data linked to data collected at ADRCs across the United States to catalyze studies of the ADRD exposome. The scope and size of TNS positions it as a unique resource within the ADRD field. In particular, its connection of in‐depth social exposome data to neuropathologically characterized brain tissue is highly unique.

TNS builds residential histories through life‐course data collection methods to contextualize biological aging in regionally and racially/ethnically diverse populations. While a few large studies examined the effect of the adverse social exposome on brain health,^17^, ^18^ large, multi‐site US cohorts with life‐course social exposome data linked to both brain tissue and neuroimaging outcomes in ADRD were lacking due to the massive undertaking. Indeed, TNS only became feasible by the collaboration of the ADRC network and support from the National Institute on Aging (NIA). ADRCs lead the world in clinical studies, identify risk and protective factors, and move the field forward in brain aging research. TNS builds on this remarkable ADRC network resource to further the understanding of the social exposome, allowing the development of new precision prevention interventions for ADRD risk mitigation.

While each individual ADRC has a swath of clinical, biological, and imaging data, to fully understand how the social exposome can affect ADRD risk, multi‐site studies are necessary to optimize generalizability and to provide adequate power to assess novel mechanistic pathways. TNS also leverages strategic communication platforms to build a consortium of interested ADRD exposome researchers and to curate a community of scientific collaboration to forward the ADRD exposome field.

TNS links metrics of life‐course exposome to well‐characterized ADRD‐focused biological outcomes including neuroimaging and neuropathology. TNS includes thousands of participants with volumetric magnetic resonance imaging, amyloid and tau positron emission tomography imaging, information on cerebrovascular pathology, Lewy body disease, and limbic‐predominant aging‐related TDP‐43 encephalopathy neuropathologic change (LATE‐NC), as well as numerous other outcomes. The ADRD field can leverage these outcome data to understand the intersection of adverse social exposome, comorbidities like heart disease and diabetes, like those found in the UDS and MDS datasets, and individual‐level social factors, including education, to better understand the interplay among these factors and ADRD risk. This collaborative work will also forward efforts on risk identification for non‐amyloid dementias, more common in those from disadvantaged backgrounds,^19^ and is imperative for the development of novel risk mitigation interventions for these neurodegenerative conditions.

The unique TNS life‐course data offer interested researchers an exceptional opportunity to better understand how the exposome at precise times across a person's life can affect brain health and ADRD. Critical windows of adverse social exposome exposure across the life course may lead to increased risk (or resilience), yet little is known regarding such timed exposures and ADRD risk.^20^ Most studies that offer in‐depth markers of brain health lack life‐course perspectives. Adverse exposome is more often experienced by persons who reside in disadvantaged areas or those who identify as ethnic or racial minorities; populations who also experience greater ADRD risk.^19^, ^21^ Yet, these disproportionately impacted populations may not receive accurate diagnoses or treatment as they may not have access to necessary care and may be more likely to have non‐amyloid presenting dementia.^19^, ^22^, ^23^ The hope is that eventually, with additional research, clinicians may use what they know of a patient's life course to potentially screen for atypical phenotypes earlier. To realize this promise will require greater understanding of how one's life course may shape disease presentation and phenotype, which the TNS infrastructure is poised to advance.

The promise of TNS archival data documenting brain donors’ full life course is also profound. With rigorous, reliable, and validated methods leveraging archival data, researchers can glean new and critical information to link life‐course social exposome to well‐phenotyped brain bank samples, unlocking new windows for understanding precise ADRD pathways of risk.

TNS is committed to the development and the free use of scientific data, including the ADI, to better understand the social exposome's role in health disparities research. In addition to the use of the ADI in ADRD‐related science, TNS is committed to dissemination and free access of the ADI to the public. More than 16,000 entities, including a variety of private and public bodies, use the ADI to inform their work. For example, the City of Milwaukee currently uses the ADI to determine priorities on lead pipe removal and replacement, resulting in > 10,000 households in high ADI areas receiving new pipes to date. Further, the Neighborhood Atlas website, which disseminates ADI to all, has had > 1 million views to date. The Neighborhood Atlas site will continue to serve as a data democratization platform to the general public, providing access to the ADI and other future measures of adverse social exposome as they are developed and tested. The Neighborhood Atlas can be found at https://www.neighborhoodatlas.medicine.wisc.edu/.

While TNS is at the forefront of exposomal research in ADRD, it is not without its limitations. Inherently, ADRC locations tend to be in large, urban dwelling academic centers, like the ones in this study.^24^ Most recruit participants from geographically proximate locations leading to an under‐representation of certain US populations and geographies. However, the multi‐site nature of TNS, which includes a majority of the ADRC network, does optimize geographic and population diversity to the extent possible within the network as a whole. Future work will continue to build additional avenues toward population generalizability. Last, the ADI is not the only measure of the social exposome and does not fully encompass every exposure a person may encounter; however, it does include 17 factors across four domains and has been validated for use with ADRD and a wide variety of other health outcomes. In the future with continued funding, TNS will add additional social exposome metrics in conjunction with the ADI to allow for a more complete picture of ADRD life‐course risk.

In conclusion, TNS is the largest study of its kind on social exposome in ADRD, building upon the considerable investments made into an unparalleled ADRC national network. This study provides important infrastructure that will be vital to facilitate ADRD exposome science, while simultaneously building a scientific consortium toward new knowledge. Its findings will directly inform new precision prevention approaches that can be used by clinicians, researchers, and policy makers to help mitigate ADRD. Much is yet to come for TNS across the ADRD research landscape.

## CONFLICT OF INTEREST STATEMENT

Dr. Apostolova: NIH, Life Molecular Imaging, Alzheimer Association, Roche Diagnostics, AVID Pharmaceuticals, Eli Lilly, Biogen, GE Healthcare, Prothena, Eisai, IQVIA, Genentech, Alnylam, Siemens, Otsuka, Corium. Dr. Mecca: National Institutes of Health, Genentech, Janssen, and Eli Lilly outside the submitted work. Dr. Kind: Funding from NIH and travel support from Alzheimer's Association. Dr. van Dyck: National Institutes of Health, Eli Lilly, Eisai, Roche, Genentech, Biogen, UCB, and Cerevel, and consultancies with BMS, Eisai, Cerevel, and UCB. Drs. George, Keller, Abner, Adar, Alosco, Bakulski, Barnes, Bateman, Batterman, Beach, Bendlin, Bennett, Betthauser, Brewer, Buckingham, Carrion, Chodosh, Craft, Croff, Fabio, Farias, Feldman, Goldstein, Goutman, Green‐Harris, Henderson, Kofler, Kucharska‐Newton, Lamar, Lanata, Lepping, Lingler, Lockhart, Mahnken, Marsh, Mathew, Meyer, Miller, Morris, Neugroschl, O'Connor, Paulson, Perrin, Pettigrew, Pierce, Powell, Pyarajan, Raji, Reiman, Risacher, Rissman, Rodriguez Espinoza, Sano, Saykin, Serrano, Singh, Soldan, Sultzer, van Dyck, Whitmer, Wisniewski, Woltjer, Yu, and Zhu do not have any disclosures. Author disclosures are available in the supporting information.

## CONSENT STATEMENT

For the living cohort, each eligible participant was identified by their ADRC and informed consent was provided for all participants via their institution's institutional review boards. For the brain bank cohort, all participants included were brain donors and participation followed each site's protocols for brain donation. Informed consent was not required, as this study was deemed to not be human subjects research by each institution's institutional review boards.

## Supporting information



Supporting Information

## References

[1] GBD 2019 Dementia Forecasting Collaborators . Estimation of the global prevalence of dementia in 2019 and forecasted prevalence in 2050: an analysis for the Global Burden of Disease Study 2019. Lancet Public Health. 2022;7(2):e105‐e125. doi:10.1016/S2468-2667(21)00249-8 34998485 PMC8810394

[2] Livingston G , Huntley J , Liu KY , et al. Dementia prevention, intervention, and care: 2024 report of the Lancet standing Commission. Lancet. 2024;404(10452):572‐628. doi:10.1016/S0140-6736(24)01296-0 39096926

[3] Senier L , Brown P , Shostak S , Hanna B . The socio‐exposome: advancing exposure science and environmental justice in a post‐genomic era. Environ Sociol. 2017;3(2):107‐121. doi:10.1080/23251042.2016.1220848 28944245 PMC5604315

[4] Hunt JFV , Buckingham W , Kim AJ , et al. Association of neighborhood‐level disadvantage with cerebral and hippocampal volume. JAMA Neurol. 2020;77(4):451‐460. doi:10.1001/jamaneurol.2019.4501 31904767 PMC6990953

[5] Kim B , Yannatos I , Blam K , et al. Neighborhood disadvantage reduces cognitive reserve independent of neuropathologic change. Alzheimers Dement. 2024;20(4):2707‐2718. doi:10.1002/alz.13736 38400524 PMC11032541

[6] Powell WR , Buckingham WR , Larson JL , et al. Association of neighborhood‐level disadvantage with Alzheimer disease neuropathology. JAMA Netw Open. 2020;3(6):e207559. doi:10.1001/jamanetworkopen.2020.7559 32525547 PMC7290421

[7] Wong CG , Miller JB , Zhang F , et al. Evaluation of neighborhood‐level disadvantage and cognition in Mexican American and Non‐Hispanic White adults 50 Years and older in the US. JAMA Netw Open. 2023;6(8):e2325325. doi:10.1001/jamanetworkopen.2023.25325 37647071 PMC10469291

[8] Keller SA , DeWitt A , Powell WR , et al. Adverse social exposome over the life course and vascular brain injury. JAMA Netw Open. 2025;8(5):e2512289. 40423969 10.1001/jamanetworkopen.2025.12289 PMC12117465

[9] Hayes‐Larson E , Mobley TM , Mungas D , et al. Accounting for lack of representation in dementia research: generalizing KHANDLE study findings on the prevalence of cognitive impairment to the California older population. Alzheimers Dement. 2022;18(11):2209‐2217. doi:10.1002/alz.12522 35102726 PMC9339583

[10] Mueller SG , Weiner MW , Thal LJ , et al. The Alzheimer's disease neuroimaging initiative. Neuroimaging Clin N Am. 2005;15(4):869‐877. doi:10.1016/j.nic.2005.09.008 16443497 PMC2376747

[11] Beekly DL , Ramos EM , Lee WW , et al. The National Alzheimer's Coordinating Center (NACC) database: the Uniform Data Set. Alzheimer Dis. 2007;21(3):249‐258. doi:10.1097/WAD.0b013e318142774e 17804958

[12] Melcher EM , Vilen L , Pfaff A , et al. Deriving life‐course residential histories in brain bank cohorts: a feasibility study. Alzheimers Dement. 2024;20(5):3219‐3227. doi:10.1002/alz.13773 38497250 PMC11095419

[13] Link BG , Phelan J . Social conditions as fundamental causes of disease. J Health Soc Behav. 1995;80‐94. doi:10.2307/2626958. Published online. 7560851

[14] Morris JC , Weintraub S , Chui HC , et al. The Uniform Data Set (UDS): clinical and cognitive variables and descriptive data from Alzheimer Disease Centers. Alzheimer Dis. 2006;20(4):210. doi:10.1097/01.wad.0000213865.09806.92 17132964

[15] Kind AJH , Buckingham WR . Making neighborhood‐disadvantage metrics accessible — the neighborhood atlas. N Engl J Med. 2018;378(26):2456‐2458. doi:10.1056/NEJMp1802313 29949490 PMC6051533

[16] Besser LM , Kukull WA , Teylan MA , et al. The revised National Alzheimer's Coordinating Center's Neuropathology Form‐available data and new analyses. J Neuropathol Exp Neurol. 2018;77(8):717‐726. doi:10.1093/jnen/nly049 29945202 PMC6044344

[17] Adams RJ , Howard N , Tucker G , et al. Effects of area deprivation on health risks and outcomes: a multilevel, cross‐sectional, Australian population study. Int J Public Health. 2009;54(3):183‐192. doi:10.1007/s00038-009-7113-x 19214382

[18] Bakolis I , Murray ET , Hardy R , Hatch SL , Richards M . Area disadvantage and mental health over the life course: a 69‐year prospective birth cohort study. Soc Psychiatry Psychiatr Epidemiol. 2023;58(5):735‐744. doi:10.1007/s00127-023-02427-x 36757437 PMC10097760

[19] Babulal GM , Quiroz YT , Albensi BC , et al. Perspectives on ethnic and racial disparities in Alzheimer's disease and related dementias: update and areas of immediate need. Alzheimers Dement. 2019;15(2):292‐312. doi:10.1016/j.jalz.2018.09.009 30555031 PMC6368893

[20] Pini L , Wennberg A . Critical windows into a changing world: taking a life course and cohort view of Alzheimer's disease and related dementias risk. Int Psychogeriatr. 2022;34(4):311‐313. doi:10.1017/S1041610221002751 35538871

[21] Chin AL , Negash S , Hamilton R . Diversity and disparity in dementia: the impact of ethnoracial differences in Alzheimer's disease. Alzheimer Dis Assoc Disord. 2011;25(3):187‐195. doi:10.1097/WAD.0b013e318211c6c9 21399486 PMC3396146

[22] Rosselli M , Uribe IV , Ahne E , Shihadeh L . Culture, ethnicity, and level of education in Alzheimer's disease. Neurotherapeutics. 2022;19(1):26‐54. doi:10.1007/s13311-022-01193-z 35347644 PMC8960082

[23] Wilkins CH , Windon CC , Dilworth‐Anderson P , et al. Racial and ethnic differences in amyloid PET positivity in individuals with mild cognitive impairment or dementia. JAMA Neurol. 2022;79(11):1139‐1147. doi:10.1001/jamaneurol.2022.3157 36190710 PMC9531087

[24] Arce Rentería M , Mobley TM , Evangelista ND , et al. Representativeness of samples enrolled in Alzheimer's disease research centers. Alzheimers Dement. 2023;15(2):e12450. doi:10.1002/dad2.12450 PMC10242202 37287650

